# Arterial Stiffness, Biomarkers of Liver Fat, and the Development of Metabolic Dysfunction in Metabolically Healthy Population: A Prospective Study

**DOI:** 10.3389/fcvm.2022.928782

**Published:** 2022-06-09

**Authors:** Lin Lin, Long Wang, Rui Du, Chunyan Hu, Jieli Lu, Tiange Wang, Mian Li, Zhiyun Zhao, Yu Xu, Min Xu, Yufang Bi, Weiqing Wang, Guang Ning, Yuhong Chen

**Affiliations:** ^1^Department of Endocrine and Metabolic Diseases, Shanghai Institute of Endocrine and Metabolic Diseases, Ruijin Hospital, Shanghai Jiao Tong University School of Medicine, Shanghai, China; ^2^Shanghai National Clinical Research Center for Endocrine and Metabolic Diseases, Key Laboratory for Endocrine and Metabolic Diseases of the National Health Commission of the PR China, Shanghai National Center for Translational Medicine, Ruijin Hospital, Shanghai Jiao Tong University School of Medicine, Shanghai, China

**Keywords:** arterial stiffness, obesity, metabolic syndrome, diabetes, fatty liver

## Abstract

**Background:**

Metabolic dysfunction is known to be associated with arterial stiffness. However, the risks of metabolic syndrome and diabetes due to arterial stiffness and the potential mechanism remain unclear. We aimed to investigate the association of arterial stiffness with the risk of metabolic syndrome and diabetes, and determine whether this association is mediated by liver fat.

**Methods:**

A prospective study was conducted with 4,139 Chinese adults who were metabolically healthy at baseline. Arterial stiffness was measured by brachial–ankle pulse wave velocity (baPWV). Obesity was defined as body mass index ≥25 kg/m^2^. The primary outcomes were incident metabolic syndrome and diabetes.

**Results:**

During a median follow-up of 4.4 years, 1,022 (24.7%) and 354 (9.5%) participants developed metabolic syndrome and diabetes, respectively. Compared with those in the lowest quartile of baPWV, participants in the highest quartile had 85 and 91% higher risks of metabolic syndrome and diabetes [risk ratio (RR) 1.85, 95% confidence interval (CI) 1.41, 2.42 for metabolic syndrome; RR 1.91, 95% CI 1.16, 3.15 for diabetes]. Mediation analyses indicated that fatty liver significantly mediated the association of arterial stiffness with metabolic syndrome and diabetes risk. Specifically, 18.4% of metabolic syndrome and 12.6% of diabetes risk due to arterial stiffness were mediated through fatty liver.

**Conclusions:**

Arterial stiffness was associated with higher risks of metabolic syndrome and diabetes in individuals with obesity. This association may be partially mediated by fatty liver.

## Introduction

Metabolic syndrome has dramatically increased over recent decades and has become a major global health problem (1–3). Characterized by a constellation of medical disorders, metabolic syndrome contributes to an increased risk of cardiovascular disease (CVD) and diabetes morbidity (4). As one of the most important components and complications of the metabolic syndrome, diabetes has become a major cause of cardiovascular mortality and disability worldwide (5–7). It has been estimated that 113.9 million Chinese adults had diabetes and 493.4 million had prediabetes in 2010 (8). This emphasizes the need to identify potential risk markers beyond the established ones, such as older age and unhealthy lifestyle, for early detection and risk stratification of the metabolic syndrome and diabetes.

Arterial stiffness is caused by detrimental structural and functional changes in the arterial walls. Brachial-ankle pulse wave velocity (baPWV) is a widely validated, non-invasive measure of arterial stiffness that is used in large-scale studies (9). The risk factors for arterial stiffness include older age and cardiometabolic risk factors, such as obesity, hypertension, hyperglycemia, and dyslipidemia (10). We have recently demonstrated that metabolic deterioration in obese subjects confers an increased risk of arterial stiffness (11).

It is well accepted that increased arterial stiffness develops in the presence of metabolic syndrome and diabetes, contributing to various cardiovascular complications. However, it has been suggested that arterial stiffness is a possible risk marker for metabolic disorders and that the traditional perspective that metabolic disorders precede arterial stiffness might need to be reconsidered. Muhammad et al. (12) observed that increased arterial stiffness was associated with higher risk of type 2 diabetes. Similar results were also obtained in two Chinese studies, either with cross-sectional design (13) or among specifically hypertensive patients (14). In addition, whether baPWV, a direct measurement of arterial stiffness, has any predictive value for the risk of developing metabolic syndrome has not yet been explored.

In effect, the underlying mechanisms linking arterial stiffness to metabolic syndrome and diabetes remain to be elucidated. Accumulating evidence suggests that liver fat may promote the development of increased arterial stiffness, obesity, and cardiometabolic disorders (15). It was well accepted that individuals with obesity were more vulnerable to fatty liver (16). Prior work by Rider et al. (17) found liver fat was associated with aortic stiffness, and this association was likely to be mediated *via* increasing circulating triglyceride (TG). In addition to the association with arterial stiffness, fatty liver is thought to independently promote impaired metabolic function (18). Whether fatty liver was a mediator in the relationship between arterial stiffness and metabolic dysfunction remained unknown.

In this study, we aimed to explore whether arterial stiffness, as determined by baPWV, has any predictive value for the risk of developing metabolic syndrome and diabetes, and investigate the association of arterial stiffness with incidence of metabolic syndrome and diabetes in the overall population and obesity subgroups. The mediating effect of liver fat on this potential relationship will be further investigated.

## Materials and Methods

### Study Participants

The participants were from an ongoing cohort study that we launched in Jiading District, Shanghai, China. A detailed description of the study design, eligibility criteria, and sampling has been published previously (11, 19, 20). Between March and August 2010, 10,569 local residents aged ≥ 40 years were invited to participate by examination notice and home visits. A total of 10,375 individuals agreed to take part in the survey, with a participation rate of 98.2%. Each participant received a face-to-face interview with a detailed questionnaire. For the current analysis, individuals who met the following criteria were excluded sequentially from the analyses: (1) those with self-reported CVD at baseline (*n* = 850); (2) those with metabolic syndrome at baseline (*n* = 2,697); (3) those with missing data on baPWV (*n* = 307). During August 2014 to July 2015, 4,139 eligible participants were invited to attend a follow-up visit with excluding those who were lost to follow-up (*n* = 2,382). 402 participants with diabetes at baseline were further excluded from the diabetes analysis (Supplementary Figure 1). The study was approved by the Medical Ethics Committee of Ruijin Hospital, Shanghai Jiaotong University School of Medicine. All study participants provided written voluntary informed consent.

### Baseline Data Collection

A standard questionnaire was used by trained staff through face-to-face interviews to collect information on sociodemographic characteristics, lifestyle factors, medical history, and current use of medication. The current smoker or drinker was defined as smoking cigarettes or consuming alcohol regularly in the past 6 months. Physical activity was estimated using the short form of the International Physical Activity Questionnaire (IPAQ) and metabolic equivalent hours per week (MET-h/wk) were calculated (21).

Anthropometric measurements, including blood pressure (BP), body weight and height, and waist circumference (WC) were performed by trained study nurses according to standard protocols. Body mass index (BMI) was calculated as body weight divided by body height squared (kg/m^2^). Hepatic ultrasonic examination was performed in all participants by two trained ultrasonographers, using a high-resolution B-mode tomographic ultrasound system (Esaote Biomedica SpA, Italy) with a 3.5-MHz probe. Non-alcoholic fatty liver disease (NAFLD) was diagnosed by ultrasonography with the presence of at least two of the following findings: (1) diffusely increased echogenicity of the liver relative to the kidney or spleen; (2) ultrasound beam attenuation; or (3) poor visualization of intrahepatic structures, after excluding those of excessive alcohol consumption and other known liver disease (22, 23).

All the participants underwent baPWV measurements, with baPWV values determined by Colin VP-1000 device (Model BP203RPE II, form PWV/ABI; Omron Colin Medical Instruments, Tokyo, Japan). baPWV of each side was calculated as the distance divided by the delayed time. The mean value of right and left baPWV was used for analysis.

Venous blood samples were collected after at least 10 h of overnight fasting and post-load glucose samples were acquired during a standard 75 g oral glucose tolerance test (OGTT). Plasma glucose was measured on an autoanalyzer (Modular P800; Roche, Basel, Switzerland) using the glucose oxidation method. Total cholesterol, high-density lipoprotein cholesterol (HDL-C), low-density lipoprotein cholesterol (LDL-C), TG, serum insulin and liver enzymes such as alanine aminotransferase (ALT) and aspartate aminotransferase (AST) were also measured on an autoanalyzer (Modular E170; Roche, Basel, Switzerland) using the chemiluminescence method. The total and differential white blood cell (WBC) counts were determined by an automated blood cell counter (ABX-120 system, France). Blood was shipped at 2–8°C to a certified central laboratory at Rui-Jin Hospital, Shanghai, China (certificated by the US National Glycohemoglobin Standardization Program and passed the Laboratory Accreditation Program of the College of American Pathologists). The homeostasis model assessment of insulin resistance (HOMA-IR) was calculated as fasting insulin (μU/ml) × fasting plasma glucose (FPG) (mg/dl)/405 (24). β-Cell function was estimated by the HOMA of β-cell function (HOMA-B) index: [360 × fasting insulin (μU/ml)]/[FPG (mg/dl)-63] (24). Fatty liver index (FLI) is a non-invasive method of assessing hepatic steatosis and is calculated by the following formula (25): FLI = [e ^0.953*loge(*triglycerides*) +0.139*BMI + 0.718*loge(*GGT*) + 0.053*waist circumference−15.745^] / [1 + e ^0.953*loge(*triglycerides*) + 0.139*BMI^+ ^0.718*loge(*GGT*) + 0.053*waist circumference−15.745^] ^*^ 100.

### Follow-Up and Outcome Assessment

From August 2014 to July 2015, all the eligible participants were invited to attend a follow-up visit. Data were re-collected using similar study protocols and study procedures to the baseline examination to evaluate lifestyle factors and medical history. Blood and urine samples were obtained using the same protocols that were used during the baseline examination. OGTT was also performed for each participant at follow-up visit to identify new-onset diabetes.

The metabolic syndrome was defined according to the US National Cholesterol Education Program Adult Treatment Panel III (NCEP ATP III) criteria (26). Incident metabolic syndrome was defined as having three or more of the following metabolic risk factors: (1) central obesity (WC ≥ 88 cm in women and ≥ 102 cm in men); (2) elevated systolic blood pressure (SBP) (≥ 130 mmHg) and/or diastolic blood pressure (DBP) (≥ 85 mmHg), or on antihypertensive treatment; (3) high triglyceride (≥ 1.7 mmol/l) or on lipid-lowering medications; (4) high FPG (≥ 5.6 mmol/l), or on medications for lowering glucose; and (5) low HDL-C (<1.04 mmol/l in men and <1.29 mmol/l in women) (27). Incident diabetes was defined as a FPG ≥ 7.0 mmol/L or 2-h post-load glucose ≥ 11.1 mmol/L, or a self-reported previous diagnosis by health care at a follow-up visit among participants without diabetes at baseline.

### Statistical Analysis

The baseline characteristics of participants according to incident metabolic syndrome (yes/no) were described as mean ± SD for the normally distributed continuous variables, median (interquartile range) for the skewed continuous variables, or numbers (proportions) for categorical variables. The analyses of continuous and categorical variables to assess differences among the two groups were determined by one-way analysis of variance or the χ2 test.

The levels of baPWV were categorized by sex-specific quartiles of <1,244; 1,244–1,354.3; 1,354.4–1,509; or >1,509 cm/s in men, and of <1,199.5; 1,199.5–1,315.5; 1,315.6–1,458; or >1,458 cm/s in women. We used relative risk regression analyses to examine the associations of baseline baPWV quartiles with incident metabolic syndrome and diabetes in overall and obesity participants (BMI ≥ 25 kg/m^2^). The risk ratio (RR) and 95% confidence interval (CI) of incident metabolic syndrome and diabetes with each 1-SD) increment of baPWV and for participants in the upper quartile of baPWV, compared with other participants were also evaluated through relative risk regression analyses. Tests for interactions were also conducted with including simultaneously obesity status, baseline baPWV quartiles and the respective interaction terms (obesity status multiplied by baPWV quartiles) in the models.

We examined potential does-response associations between the levels of baseline baPWV and the incidence of metabolic syndrome and diabetes (28). The baPWV was coded using a restricted cubic spline (RCS) function with three knots, located at the 5^th^, 50^th^, and 95^th^ percentiles of the distribution of baPWV. Tests for non-linearity, which compared a model containing only the linear term with a model containing the linear and restricted cubic spline terms, were performed using likelihood ratio tests. If a test for non-linearity was not significant, a test for linearity was performed, comparing a model containing the linear term with a model containing only the covariates of interest. Models were adjusted for several cofactors including age, sex, current smoking, current drinking, education, physical activity, SBP, BMI, LDL-C, and FPG.

Mediation analysis was performed to estimate the portion of the total effect of baPWV on metabolic syndrome and diabetes risk mediated by biomarkers of liver fat (NAFLD status, FLI, liver enzymes, lipid profiles, WBC, HOMA-IR, or HOMA-B). Linear regression or logistic regression was used to determine biomarkers that were associated with baPWV. Relative risk regression models using the biomarkers as the exposure, and metabolic syndrome and diabetes as the outcome were examined. These regressions were integrated to obtain the effect mediated by a specific biomarker, to calculate the proportion mediated and test its significance. All models were adjusted for age, sex, current smokers (yes/no), current drinking (yes/no), education, physical activity. Direct effects, indirect effects, their 95% CIs, and the mediated proportions were obtained using the R package for mediation analyses.

Significance tests were two-tailed, with *P* < 0.05 considered as statistically significant. We used R version 4.0.1 (R Foundation for Statistical Computing, Vienna, Austria) to perform mediation analyses and SAS version 9.4 (SAS Institute, Cary, NC, USA) to conduct the remaining statistical analyses.

## Results

### Baseline Characteristics of the Study Population

Baseline sociodemographic and biochemical characteristics of the participants according to incident metabolic syndrome status during follow-up are shown in Table 1. Participants with incident metabolic syndrome were more likely to be female, older, less educated, and obese. The levels of BP, FPG, TG, total cholesterol, LDL-C, ALT, and HOMA-IR were higher, and HDL-C was lower in those case participants with incident metabolic syndrome. They had higher usage of antidiabetic and antihypertensive drugs. The levels of baPWV were found to be higher in individuals who developed incident metabolic syndrome than those without.

**Table 1:** Baseline characteristics of participants according to incident metabolic syndrome (*n* = 4,139).

**Characteristics**	**Metabolic syndrome (-) (*n* = 3,117)**	**Metabolic syndrome (+) (*n* = 1,022)**	***P-*value**
Male, *n* (%)	1,357 (43.5)	307 (30.0)	<0.0001
Age (years)	57.10 ± 8.73	57.90 ± 8.68	0.02
BMI (kg/m^2^)	24.06 ± 2.80	25.54 ± 2.79	<0.0001
Waist circumference (cm)	79.60 ± 8.08	82.95 ± 7.38	<0.0001
High school education or above, *n* (%)	698 (22.48)	173 (17.01)	0.0002
Lifestyle factors			
Current smokers, *n* (%)	744 (24.69)	176 (17.92)	<0.0001
Current drinkers, *n* (%)	356 (11.82)	103 (10.38)	0.22
Physical activity (MET-h/wk)	15.00 (0–35.00)	20.30 (0–36.00)	0.98
Blood pressure (mmHg)			
SBP	135.60 ± 19.08	142.97 ± 19.47	<0.0001
DBP	80.91 ± 9.91	83.82 ± 10.22	<0.0001
Lipid profiles (mmol/L)			
TG	1.11 (0.84–1.46)	1.40 (1.09–1.68)	<0.0001
TC	5.26 ± 0.94	5.44 ± 1.01	<0.0001
HDL-C	1.45 ± 0.32	1.32 ± 0.27	<0.0001
LDL-C	3.13 ± 0.81	3.34 ± 0.86	<0.0001
FPG (mmol/L)	5.17 ± 1.03	5.42 ± 1.14	<0.0001
HOMA-IR	1.26 (0.85–1.76)	1.66 (1.16–2.33)	<0.0001
ALT (IU/L)	19.83 ± 11.30	21.84 ± 13.01	<0.0001
AST (IU/L)	22.66 ± 7.93	22.95 ± 7.93	0.31
baPWV (cm/s)	1,476.54 ± 304.14	1,593.07 ± 348.26	<0.0001
Diabetes, *n* (%)	261 (8.37)	141 (13.80)	<0.0001
Hypertension, *n* (%)	1,347 (43.28)	651 (63.89)	<0.0001
Hypercholesterolaemia, *n* (%)	455 (14.60)	209 (20.45)	<0.0001
Drug use, n (%)			
Antidiabetic drug use	105 (3.37)	54 (5.28)	0.006
Antihypertensive drug use	541 (17.36)	320 (31.31)	<0.0001
Lipid-lowering drug use	1 (0.03)	1 (0.10)	0.41

*Data were means ± SD or medians (interquartile ranges) for skewed variables or numbers (proportions) for categorical variables. P values were calculated from one-way ANOVA for continuous variables and χ2 test for categorical variables*.

*BMI, body mass index; SBP, systolic blood pressure; DBP, diastolic blood pressure; PP, pulse pressure; TC, total cholesterol; TG, triglycerides; HDL-C, high- density lipoprotein cholesterol; LDL-C, low-density lipoprotein cholesterol; FPG, fasting plasma glucose; HOMA-IR, homeostasis model assessment of insulin resistance; baPWV, brachial-ankle pulse wave velocity*.

### Baseline baPWV and Incidence of Metabolic Syndrome and Diabetes

During up to 5 years of follow-up (mean 4.4 years), 1,022 incident cases of metabolic syndrome were counted among 4,139 participants without a history of metabolic syndrome, and 354 incident cases of diabetes were counted among 3,737 participants without a history of metabolic syndrome and diabetes.

**Figure 1:**
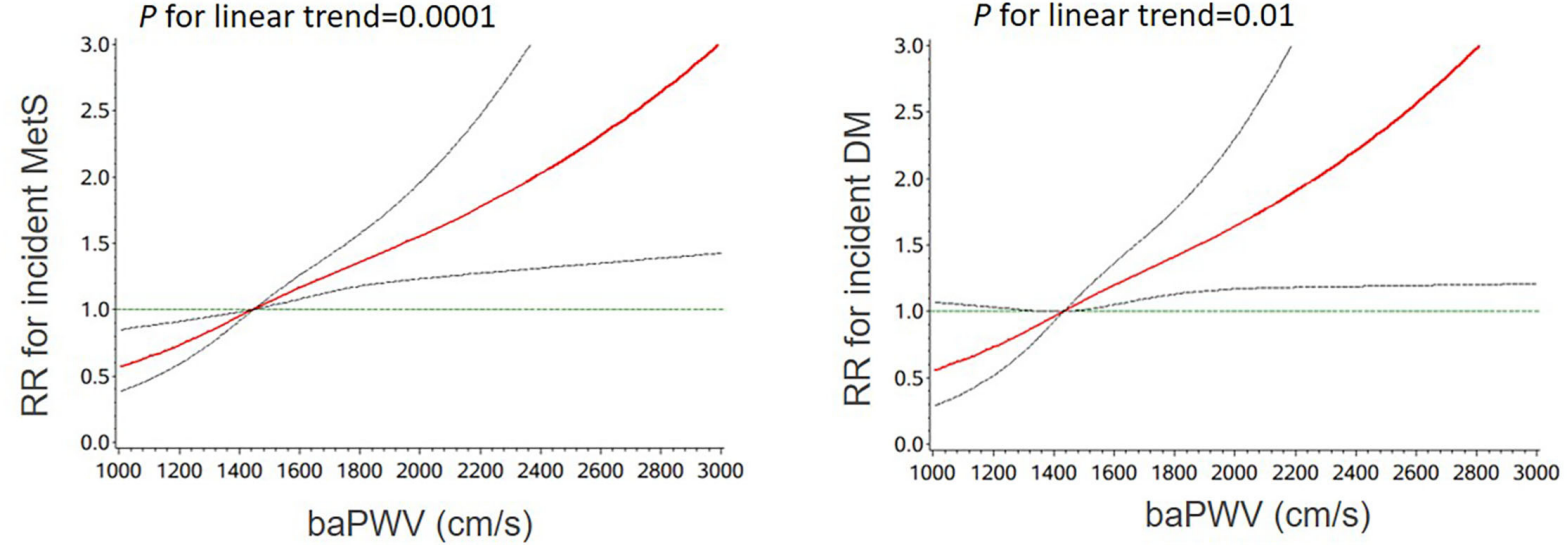
Multivariable-adjusted RRs (95% CIs) for the association of incident metabolic syndrome and diabetes with baPWV. Adjusted for age, sex, current smoking (yes/no), current drinking (yes/no), education, physical activity, SBP, BMI, LDL-C, and FPG. RR, risk ratio; CI, confidence interval; baPWV, brachial–ankle pulse wave velocity; SBP, systolic blood pressure; BMI, body mass index; LDL-C, low-density lipoprotein cholesterol; FPG, fasting plasma glucose.

Table 2 presents the risk of incident metabolic syndrome and diabetes according to baseline baPWV levels. Increased baPWV levels were significantly associated with incident metabolic syndrome and diabetes. The participants in the fourth quartile (Q4) of baPWV had a significantly higher risk of metabolic syndrome (RR 1.85, 95% CI 1.41–2.42) and diabetes (RR 1.91, 95% CI 1.16–3.15) as compared with those in the first quartile, after adjusting for important covariates. When participants in the first to third quartiles were taken as reference, the multivariable-adjusted RR (95% CI) of incident metabolic syndrome and diabetes associated with the highest quartile of baPWV was 1.42 (1.18–1.70) and 1.45 (1.28–2.02), respectively. The multivariable-adjusted RRs (95% CIs) of incident metabolic syndrome and diabetes associated with each 1-SD increment of baPWV were 1.32 (1.15–1.49) and 1.31 (1.10–1.57), respectively. Multivariable-adjusted restricted cubic spline analyses suggested significant linear relationships between baseline baPWV and both metabolic syndrome (*P* for linear trend = 0.0001) and diabetes (*P* for linear trend = 0.01) (Figure 1).

**Table 2:** RRs (95% CIs) of incident metabolic dysfunction according to baPWV levels in metabolically healthy participants.

	**Baseline baPWV Quartiles**				**RR for Q4, with Q1–Q3 as reference**	**Per SD increment of baPWV**
	**Q1**	**Q2**	**Q3**	**Q4**		
**Metabolic syndrome**						
Cases/*n* (%)	161 (15.5)	214 (20.7)	283 (27.3)	364 (35.2)	/	/
Model 1	1.00	1.33 (1.11–1.78)	1.76 (1.48–2.09)	2.27 (1.92–2.67)	1.66 (1.49–1.85)	1.41 (1.32–1.51)
Model 2	1.00	1.28 (1.00–1.63)	1.66 (1.31–2.11)	2.37 (1.87–3.00)	1.74 (1.48–2.04)	1.47 (1.32–1.64)
Model 3	1.00	1.18 (0.92–1.50)	1.45 (1.14–1.85)	1.85 (1.41–2.42)	1.42 (1.18–1.70)	1.32 (1.15–1.49)
**Diabetes**						
Cases/*n* (%)	59 (6.3)	73 (7.9)	88 (9.4)	134 (14.4)	/	/
Model 1	1.00	1.25 (0.90–1.74)	1.50 (1.09–2.06)	2.29 (1.71–3.07)	1.83 (1.50–2.24)	1.38 (1.25–1.52)
Model 2	1.00	1.19 (0.75–1.89)	1.24 (0.78–1.97)	2.04 (1.31–3.17)	1.75 (1.28–2.40)	1.32 (1.13–1.54)
Model 3	1.00	1.15 (0.72–1.84)	1.20 (0.74–1.95)	1.91 (1.16–3.15)	1.45 (1.28–2.02)	1.31 (1.10–1.57)

*Model 1, unadjusted; Model 2, adjusted for age, sex, current smoking (yes/no), current drinking (yes/no), education and physical activity. Model 3, further adjusted for SBP, BMI, LDL-c, and FPG based on model 2*.

*RR, risk ratio; CI, confidence interval; baPWV, brachial–ankle pulse wave velocity; SD, standard deviation; SBP, systolic blood pressure; BMI, body mass index; LDL-c, low-density lipoprotein cholesterol; FPG, fasting plasma glucose*.

### Stratified Analysis by Obesity Status

**Figure 2:**
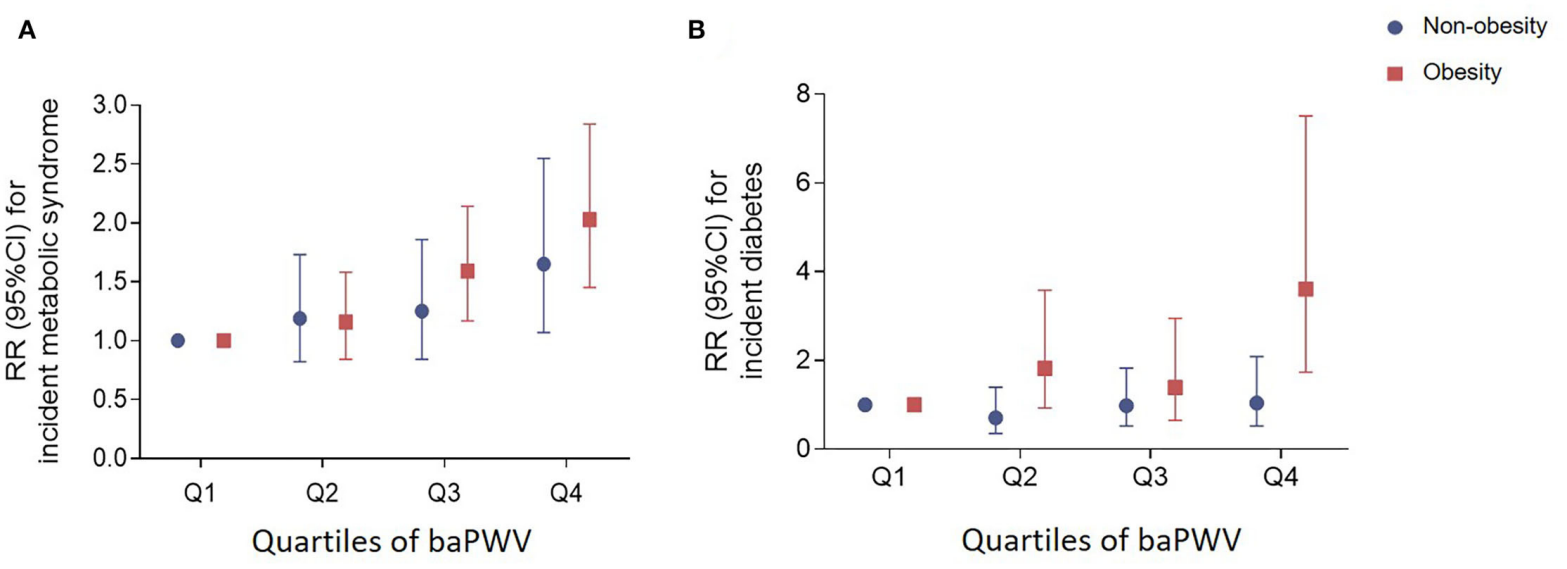
RRs (95% CIs) of incident metabolic syndrome **(A)** and diabetes **(B)** according to baPWV in non-obese and obese participants. Adjusted for age, sex, current smoking (yes/no), current drinking (yes/no), education, physical activity, SBP, BMI, LDL-C, and FPG. RR, risk ratio; CI, confidence interval; baPWV, brachial–ankle pulse wave velocity; SBP, systolic blood pressure; BMI, body mass index; LDL-C, low-density lipoprotein cholesterol; FPG, fasting plasma glucose.

We investigated whether the risks of incident metabolic syndrome and diabetes associated with baseline baPWV were modified by obesity status. The study population was stratified into those with BMI <25 kg/m^2^ and those with a BMI ≥ 25 kg/m^2^. The risks of developing metabolic syndrome associated with elevated baPWV were significantly higher in individuals with obesity [RR (95% CI) in the third and fourth quartile of baPWV: 1.59 (1.17–2.14) and 2.03 (1.45–2.84) (Figure 2). Similar results were also observed for incident diabetes (RR [95% CI] in the fourth quartile of baPWV: 3.61 (1.74–7.51)] (Figure 2). For both incident metabolic syndrome and diabetes, we observed significant differences in BMI (both *P* < 0.001 for interaction) across strata. We also compared metabolic characteristics except the components of metabolic syndrome and diabetes between the participants with normal weight and those with obesity, and found that the prevalence of NAFLD and the levels of FLI, liver enzymes, WBC count, HOMA-IR, and HOMA-B were significantly higher in the obese group (Supplementary Table 1).

### Mediation Analysis

**Figure 3:**
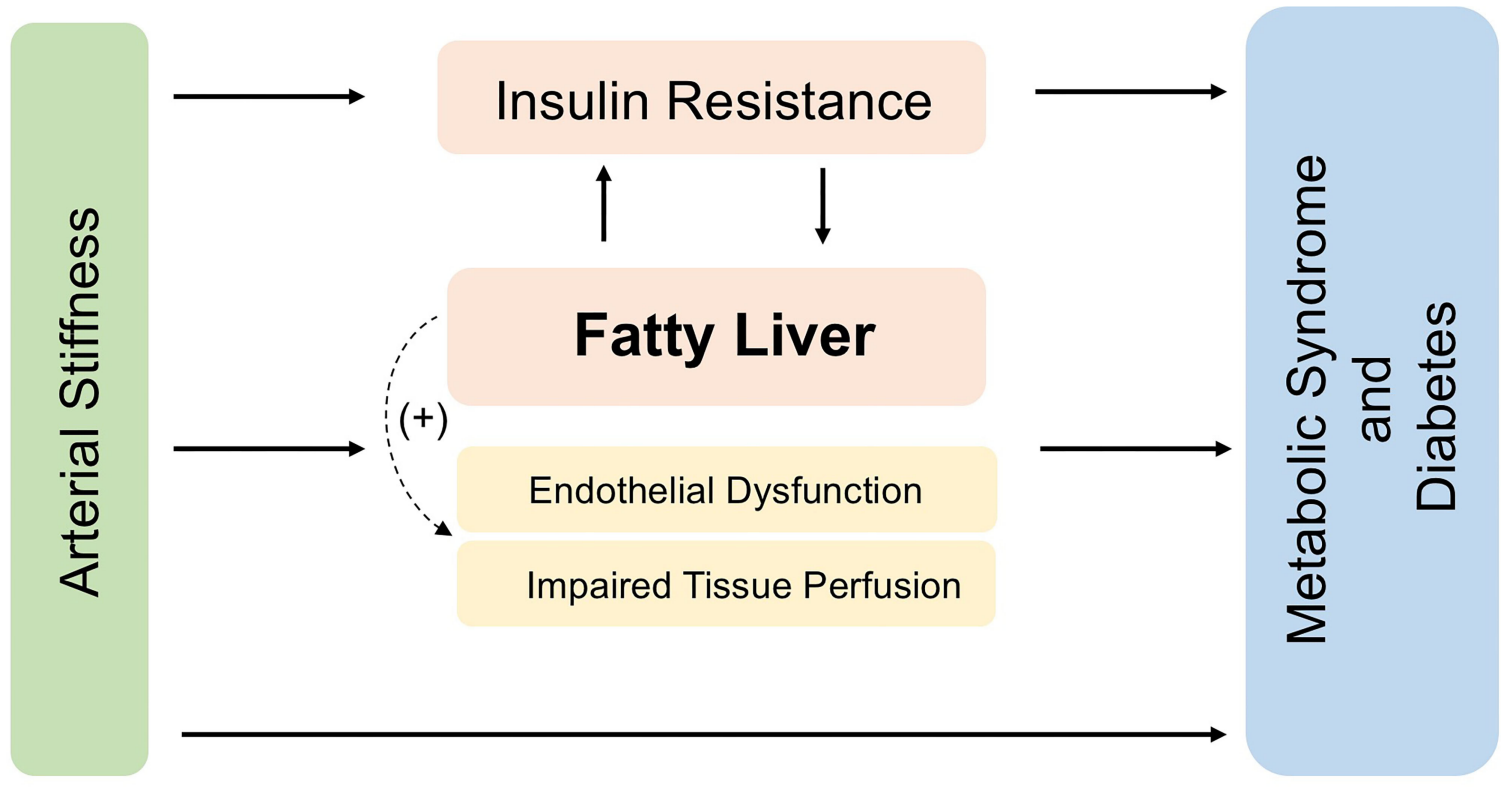
Diagram hypothesized for mediation, characterizing the relationship between arterial stiffness and metabolic dysfunction.

Biomarkers that were significantly higher in the obesity group were further examined through mediation analysis (Table 3). NAFLD, FLI, and ALT significantly mediated the baPWV-metabolic syndrome association, accounting for 18.4, 6.6, and 4.2 of the proportion, respectively. NAFLD, FLI, and ALT also significantly mediated the baPWV-diabetes association, accounting for 12.6, 8.9, and 6.8 of the proportion, respectively. HOMA-IR mediated both the baPWV-metabolic syndrome and baPWV-diabetes associations, accounting for 6.2 and 5.5% of the proportion, respectively. WBC additionally mediated the baPWV-metabolic syndrome association, accounting for 7.5% of the proportion. Diagram hypothesized for mediation, characterizing the relationship between arterial stiffness and metabolic dysfunction was presented in Figure 3.

**Table 3:** Mediation effect investigating potential factors which mediated the association of arterial stiffness with metabolic dysfunctions.

**Mediators**	**Direct effect (95% CI)**	**Indirect effect (95% CI)**	**Percent mediated (%)**
**Metabolic syndrome**			
NAFLD	0.069 (0.052–0.080)	0.007 (0.004–0.011)	18.4
FLI	0.060 (0.043–0.080)	0.014 (0.010–0.020)	6.6
ALT	0.069 (0.050–0.090)	0.003 (0.001–0.005)	4.2
AST	0.073 (0.052–0.090)	0.0002 (−0.0004–0.0005)	0.2
TG	0.072 (0.050–0.090)	0.0005 (0.0001–0.0008)	6.6
HDL-C	0.068 (0.049–0.093)	0.0003 (0.00009–0.0006)	5.7
LDL-C	0.038 (0.022–0.071)	0.005 (−0.0002–0.011)	1.2
WBC	0.067 (0.047–0.080)	0.006 (0.003–0.010)	7.5
LYM	0.070 (0.050–0.090)	0.00007 (−0.0006–0.00009)	0.09
NEU	0.070 (0.052–0.090)	0.0003 (−0.0003–0.0005)	0.3
HOMA-IR	0.067 (0.050–0.080)	0.004 (0.002–0.008)	6.2
HOMA-B	0.076 (0.057–0.090)	−0.003 (−0.006–0.0002)	4.0
**Diabetes**			
NAFLD	0.017 (0.006–0.030)	0.002 (0.0007–0.004)	12.6
FLI	0.019 (0.010–0.030)	0.001 (0.0008–0.003)	8.3
ALT	0.017 (0.006–0.030)	0.001 (0.0005–0.003)	6.8
AST	0.019 (0.011–0.030)	0.0002 (−0.0004–0.0006)	0.7
TG	0.022 (0.009–0.030)	0.0001 (−0.0006–0.0003)	2.2
HDL-C	0.060 (0.039–0.079)	0.0004 (0.0002–0.0006)	3.2
LDL-C	0.028 (0.030–0.067)	0.0003 (−0.008–0.0006)	0.5
WBC	0.020 (0.009–0.030)	0.0005 (−0.0005–0.0009)	2.6
LYM	0.021 (0.009–0.030)	0.00005 (−0.0006–0.0001)	0.04
NEU	0.018 (0.006–0.030)	0.0001 (−0.0002–0.0003)	0.4
HOMA-IR	0.018 (0.005–0.030)	0.001 (0.0005–0.003)	5.5
HOMA-B	0.019 (0.008–0.030)	0.0006 (−0.0006–0.0009)	3.4

*Adjusted for age, sex, current smoking (yes/no), current drinking (yes/no), education, physical activity*.

*CI, confidence interval; NAFLD, non-alcoholic fatty liver disease; FLI, fatty liver index; ALT, alanine transaminase; AST, aspartate aminotransferase; WBC, white blood cell; TG, triglyceride; HDL-C, high- density lipoprotein cholesterol; LDL-C, low-density lipoprotein cholesterol; LYM, lymphocyte; NEU, neutrophil; HOMA-IR, homeostasis model assessment of insulin resistance; HOMA-B, homeostasis model assessment of β-cell function*.

## Discussion

This population-based prospective study demonstrated that elevated baseline baPWV, a surrogate marker of arterial stiffness, is associated with the risk of incident metabolic syndrome and diabetes, after adjusting for important risk factors, suggesting that arterial stiffness is an independent risk factor for incident metabolic syndrome and diabetes. These associations were modified by BMI status. Obese participants had positive associations of baPWV with either metabolic syndrome or diabetes. According to the mediation analysis, fatty liver, and HOMA-IR, which were more severe in the obese participants, significantly mediated the association of baPWV with metabolic syndrome and diabetes.

Previous evidence confirmed that vessels were stiffer from early adulthood onward with a linear increase in baPWV during life (29). The potential reasons for the higher rate of developing arterial stiffness with increasing age include vascular aging and injurious effects of cardio-metabolic risk factors like hypertension and diabetes. In our study, however, no statistical differences between individuals with or without metabolic syndrome at baseline were observed in the increase rate of baPWV from baseline to the follow-up (data not shown). Actually, the process of arterial stiffness is accelerated in the presence of metabolic dysfunction. Unlike the traditional assumption that metabolic dysfunction precedes arterial stiffness, our study found a possibly inverse association. Until now, there have been few prior studies examining the relationship between arterial stiffness and incident metabolic dysfunctions. Muhammad et al. (12) conducted a cohort study consisting of 2,450 individuals without prevalent diabetes at baseline, and they found that carotid-femoral pulse wave velocity (cf PWV) was associated with increased incidence of diabetes. Stratified analyses indicated a significant relationship between carotid-femoral PWV and incident diabetes in individuals with impaired fasting glucose, instead of those with normal fasting glucose or glucose tolerance. Another recent study found a significant positive association between baseline baPWV and the risk of new-onset diabetes in hypertensive patients only, suggesting that diabetes might partly be a consequence of vascular impairment (14). Whether arterial stiffness has any predictive value for the risk of developing metabolic syndrome has not been explored. In this study, baPWV was used to measure arterial stiffness for each individual. Since its first launch for clinical use in the early 2000s, baPWV is a widely used method for assessing arterial stiffness in studies of large sample sizes, because of its reproducibility and simplicity. Besides, baPWV may be more easily applied in epidemiological practice than cfPWV (9). In particular, we used NCEP-ATP III criteria to define metabolic syndrome in that the criteria as one of the most commonly used definition of metabolic syndrome among previous studies, especially those conducted in Chinese participants (26, 30). Our study contributes new knowledge about the relative importance of baPWV on predicting the risk of both incident metabolic syndrome and diabetes, independent of important risk factors.

In the current study, the associations of baPWV with metabolic syndrome and diabetes was more evident in individuals with obesity than in those with normal weight (both *P* for interaction < 0.001), suggesting that BMI status modified those positive associations. Our findings were consistent with a cross-sectional study (13) that also indicated that individuals with overweight/obesity, instead of those with normal weight, had significant associations between elevated baPWV levels and diabetes risk. Another study revealed that women had higher susceptibility of arterial stiffening in obesity which predispose to hypertension (31). However, there was evidence showing a lower degree of stiffness in large arteries of overweight-obese subjects compared to their normal-weight counterparts (32). Whether BMI elevations could explain the increased risk of metabolic syndrome and diabetes associated with baPWV needs to be investigated. In our study, individuals with obesity tended to have NAFLD, and had higher levels of liver enzymes, FLI, compared with those without obesity, which was consistent with previous findings that individuals with obesity were more vulnerable to fatty liver (3). Accumulating evidence suggests that liver fat might related to arterial stiffness (4). The International Atherosclerosis Society and International Chair on Cardiometabolic Risk Working Group on Visceral Obesity have also summarized the concept for visceral adiposity as an emerging risk factor for type 2 diabetes, premature atherosclerosis and cardiovascular disease (15). As a result, we hypothesized that a major component of the observed negative effect is mediated *via* increased liver fat. Our mediation analyses showed that among several possible mediators, NAFLD mediated a significant proportion (18.4% for metabolic syndrome and 12.6% for diabetes) of the associations, indicated a possible mediating role of liver fat.

Although we have shown the mediation role of liver fat content on the above association, a direct negative effect of liver fat on aortic elastic function seems unlikely given the lack of spatial colocalization. One potential mechanism is that perturbed microvascular function caused by vascular stiffness, including impaired tissue perfusion, the propagation of increased pressure, and flow pulsations to the pancreatic bed may result in pancreatic dysfunction (33), and thus contribute to the development of diabetes and other metabolic disorders. Another possible explanation, which is supported by previous studies, is endothelial dysfunction and impaired endothelium-dependent vasodilation (34). Endothelial dysfunction was reported to be involved in the process of the development of arterial stiffness and metabolic dysfunction (35, 36). It was reported that obesity played a direct role in the secretion of proinflammatory cytokines (37), and systemic inflammation increases endothelial dysfunction. Increased levels of asymmetric dimethyl arginine, one of the endogenous antagonists of nitric oxide synthase, and circulating markers of systemic inflammation are typically observed in NAFLD patients (38). These mechanisms are supported by the clinical findings in a study observing significantly reduced flow-mediated vasodilation in NAFLD patients (39). Taken together, it can be speculated that arterial stiffness has the potential to disturb metabolic homeostasis by introducing impaired tissue perfusion and endothelial dysfunction, which may be exacerbated by NAFLD.

The current prospective study found a significant association between arterial stiffness and metabolic syndrome and diabetes. The entire study population all received standard OGTT tests both at baseline and at follow-up visit. We used mediation analysis to quantify the effects of mediators associated with type 2 diabetes. This method may help explain the association between arterial stiffness and risk of metabolic dysfunction observed in the prospective cohort by exploring relative contributions of potential pathways.

Our study had several limitations. First, the study was performed in middle-aged and elderly Chinese individuals, impeding drawing general conclusions. Second, the median follow-up duration of 4.4 years may have been insufficient to uncover the incidence of metabolic dysfunction and diabetes. Third, the potential mediators, such as NAFLD, were assessed at baseline, instead of yearly. It is unclear whether NAFLD will experience a transition during follow-up. Multiple assessments of these potential mediators may be warranted to help clarify this issue.

In conclusion, we found that arterial stiffness was associated with increased risks of metabolic syndrome and diabetes, and this association was partly mediated by liver fat. However, potential physiological mechanisms explaining the association of arterial stiffness with development of metabolic dysfunction requires further research among demographically diverse populations. Individuals may benefit from early intensive control of arterial stiffness and NAFLD to prevent the development of metabolic syndrome and diabetes.

## Data Availability Statement

The original contributions presented in the study are included in the article, and further inquiries can be directed to the corresponding author.

## Ethics Statement

This study was approved by the Medical Ethics Committee of Ruijin Hospital, Shanghai Jiaotong University. All study participants provided written informed consent to participate in this study.

## Author Contributions

YC and LL conceived and designed the study. LL and LW drafted the manuscript. LL and RD analyzed data. CH, JL, TW, ML, ZZ, YX, MX, and YB collected data. All authors have approved the final version to be published. YC, WW, and GN guarantee this work and have full access to all of the data and take responsibility for the integrity of the data. All authors have approved the final version to be published.

## Funding

This work was supported by the National Natural Science Foundation of China (Grant/Award Nos. 81900741 and 81870604) and Ministry of Science and Technology of the People's Republic of China (Grant/Award Nos. 2016YFC1304904, 2016YFC1305600, and 2016YFC1305202). YC was supported by the Shanghai Shenkang Hospital Development Center for improving the control of type 2 diabetes in the suburbs of Shanghai (16CR4020A). YB was supported by the Clinical Research Plan of SHDC (SHDC2020CR3064B).

## Conflict of Interest

The authors declare that the research was conducted in the absence of any commercial or financial relationships that could be construed as a potential conflict of interest.

## Publisher's Note

All claims expressed in this article are solely those of the authors and do not necessarily represent those of their affiliated organizations, or those of the publisher, the editors and the reviewers. Any product that may be evaluated in this article, or claim that may be made by its manufacturer, is not guaranteed or endorsed by the publisher.
