# Supplementary material for: Arterial Stiffness, Biomarkers of Liver Fat, and the Development of Metabolic Dysfunction in Metabolically Healthy Population: A Prospective Study
Source: Front Cardiovasc Med. 2022 Jun 9;9:928782. doi: 10.3389/fcvm.2022.928782 (PMC9261979; doi:10.3389/fcvm.2022.928782)
Supplement: Supplementary file 1 [file Data_Sheet_1.pdf]

Arterial stiffness, biomarkers of liver fat, and the development of metabolic dysfunction in metabolically healthy population: a prospective study

Lin Lin[1,2*]; Long Wang[1,2*]; Rui Du[1,2*]; Chunyan Hu[1,2]; Jieli Lu[1,2]; Tiange Wang[1,2]; Mian Li[1,2]; Zhiyun Zhao[1,2]; Yu Xu[1,2]; Min Xu[1,2]; Yufang Bi[1,2]; Weiqing Wang[1,2]; Guang Ning[1,2]; Yuhong Chen[1,2]

[1] Department of Endocrine and Metabolic Diseases, Shanghai Institute of Endocrine and Metabolic Diseases, Ruijin Hospital, Shanghai Jiao Tong University School of Medicine, Shanghai, China

[2] Shanghai National Clinical Research Center for Endocrine and Metabolic Diseases, Key Laboratory for Endocrine and Metabolic Diseases of the National Health Commission of the PR China, Shanghai National Center for Translational Medicine, Ruijin Hospital, Shanghai Jiao-Tong University School of Medicine, Shanghai, China

Supplemental Table 1. Characteristics of participants according to obesity status.

	Non-obesity	Obesity	*P* value
**Metabolic syndrome dataset**			
NAFLD, n (%)	160 (6.6)	454 (26.6)	< 0.0001
FLI	11.01 (5.93-19.20)	34.25 (23.12-50.50)	< 0.0001
ALT (U/L)	16.10 (12.60-21.20)	19.50 (14.90-26.40)	< 0.0001
AST (U/L)	21.30 (18.20-24.90)	21.60 (18.50-25.60)	0.005
WBC, 10^9/L	5.30 (4.50-6.20)	5.70 (4.80-6.70)	< 0.0001
HOMA-IR	1.18 (0.79-1.66)	1.61 (1.16-2.29)	< 0.0001
HOMA-B	71.50 (44.74-104.59)	87.70 (57.14-130.67)	< 0.0001
**Diabetes dataset**			
NAFLD, n (%)	127 (5.7)	359 (24.0)	< 0.0001
FLI	10.71 (5.75-18.87)	33.63 (22.77-49.79)	< 0.0001
ALT (U/L)	16.00 (12.50-21.00)	19.20 (14.65-26.00)	< 0.0001
AST (U/L)	21.40 (18.20-24.90)	21.60 (18.60-25.40)	0.14
WBC, 10^9/L	5.20 (4.40-6.20)	5.60 (4.80-6.70)	< 0.0001
HOMA-IR	1.16 (0.76-1.61)	1.56 (1.12-2.15)	< 0.0001
HOMA-B	74.24 (48.31-106.88)	91.94 (62.59-135.08)	< 0.0001

Note: Data were medians (interquartile ranges) for skewed variables or numbers (proportions) for categorical variables. P values were calculated from one-way ANOVA for continuous variables and $\chi 2$ test for categorical variables.

Abbreviations: NAFLD: non-alcoholic fatty liver disease; FLI: fatty liver index; ALT: alanine transaminase; AST: aspartate aminotransferase; WBC: white blood cell; HOMA-IR: homeostasis model assessment of insulin resistance; HOMA-B: homeostasis model assessment of β-cell function.

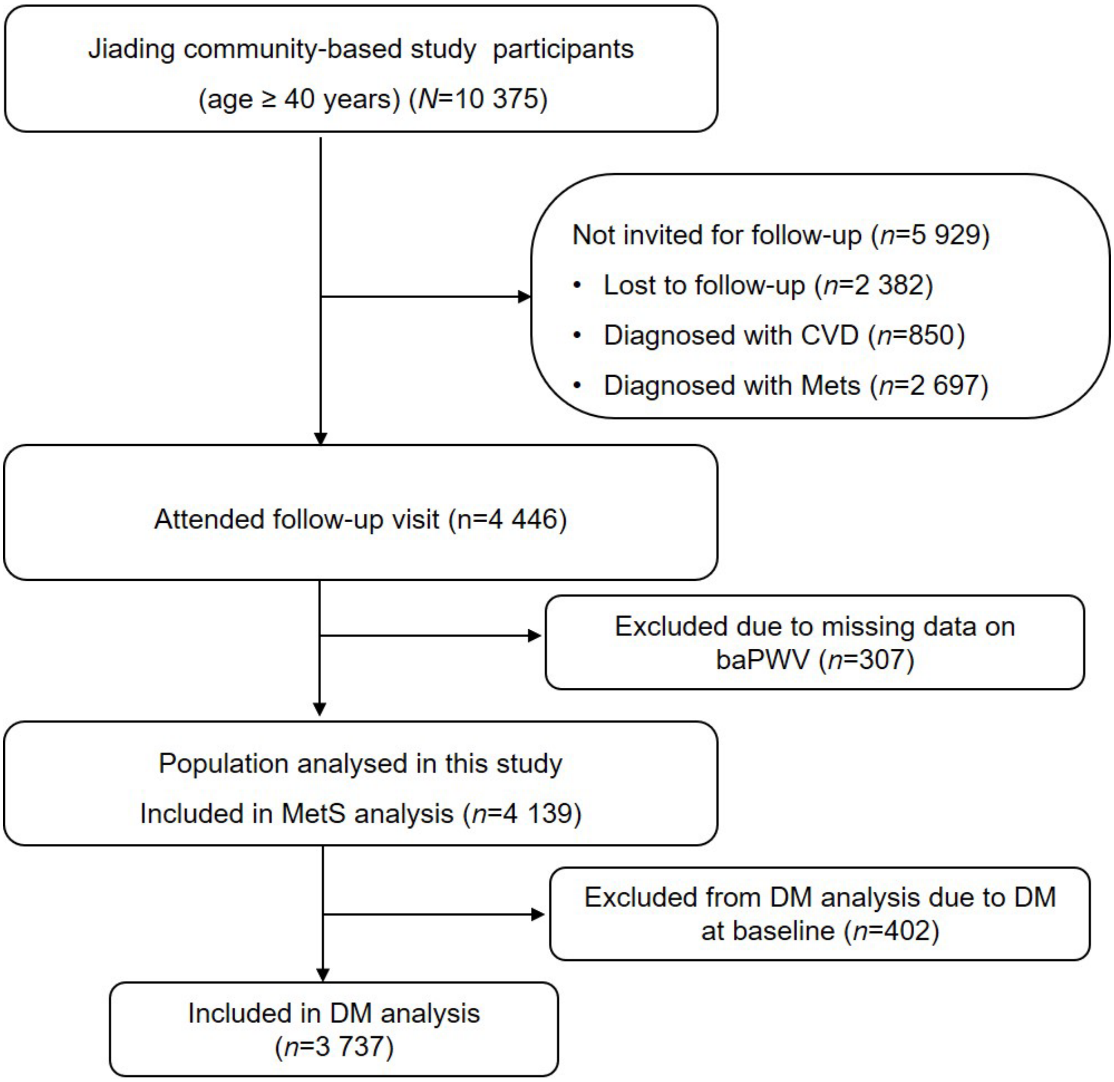

Supplemental Figure 1. Flowchart of study population. Abbreviations:CVD: cardiovascular disease; Mets: metabolic syndrome; baPWV, brachial-ankle pulse wave velocity; DM: diabetes mellitus.
